# The gut microbiome predicts response to UDCA/CDCA treatment in gallstone patients: comparison of responders and non-responders

**DOI:** 10.1038/s41598-024-53173-2

**Published:** 2024-01-30

**Authors:** Jungnam Lee, Jin-Seok Park

**Affiliations:** 1 Department of Internal Medicine, Inha University Hospital, Inha University School of Medicine, Incheon, South Korea; 2 Department of Internal Medicine, Digestive Disease Center, Shihwa Medical Center, 381, Gunjacheon-ro, Siheung-si, Gyeonggi-do South Korea

**Keywords:** Biliary tract disease, Dysbiosis

## Abstract

The treatment of gallbladder (GB) stones depends on condition severity. Ursodeoxycholic acid (UDCA) and chenodeoxycholic acid (CDCA) are commonly used to treat GB stones, but the factors affecting response rates have not been fully identified. Therefore, we investigated the relationship between response to UDCA/CDCA treatment and changes in the gut microbiomes of patients with GB stones with the intention of identifying gut microbiomes that predict susceptibility to UDCA/CDCA treatment and treatment response. In this preliminary, prospective study, 13 patients with GB stones were treated with UDCA/CDCA for 6 months. Patients were classified into responder and non-responder groups based on treatment outcomes. Gut microbiomes were analyzed by 16S rDNA sequencing. Taxonomic compositions and abundances of bacterial communities were analyzed before and after UDCA/CDCA treatment. Alpha and beta diversities were used to assess similarities between organismal compositions. In addition, PICRUSt2 analysis was conducted to identify gut microbial functional pathways. Thirteen patients completed the treatment; 8 (62%) were assigned to the responder group and the remainder to the non-responder group. Low abundances of the *Erysipelotrichi* lineage were significantly associated with favorable response to UDCA/CDCA treatment, whereas high abundances of *Firmicutes* phylum indicated no or poor response. Our results suggest that a low abundance of the *Erysipelotrichi* lineage is significantly associated with a favorable response to UDCA/CDCA and that a high abundance of *Firmicutes* phylum is indicative of no or poor response. These findings suggest that some gut microbiomes are susceptible to UDCA/CDCA treatment and could be used to predict treatment response in patients with GB stones.

## Introduction

Gallbladder (GB) stones are formed when bile components solidify within the GB, causing an imbalance in bile composition and the precipitation of cholesterol or other substances^1,2^. GB stone prevalence varies across regions but is estimated to affect approximately 10–15% of the general population in the United States. Furthermore, its prevalence tends to increase with age, obesity, and metabolic syndrome^3^. GB stones can cause discomfort and sometimes serious health problems, including sudden and rapidly intensifying pain, fever, chills, jaundice, and acute GB inflammation^1,4^.

GB stone treatment depends on condition severity^5^. Cholecystectomy is the most common treatment option for symptomatic gallstones^5^, whereas litholysis plus medical treatment is reserved for patients unsuitable for surgical intervention or at risk of surgical complications^6,7^. Ursodeoxycholic acid (UDCA) and chenodeoxycholic acid (CDCA) are bile acids naturally produced in liver from cholesterol and have been used to treat gallbladder stones^6–8^. Combined UDCA/CDCA treatment has also been used to treat or prevent several hepatic and biliary diseases, including cholestatic liver disease and primary biliary cirrhosis^9,10^. Prospective studies have reported response rates to UDCA/CDCA treatment ranging from 43.2 to 47.2%. However, with the exception of GB stone size, the factors affecting response to treatment remain unidentified^6,7,11–13^. Furthermore, no biomarker has been identified that accurately predicts response to UDCA/CDCA treatment for managing GB stones. While several criteria such as Barcelona, Paris-I/II, GLOBE, UK-PBC scores have been proposed for the evaluation of treatment response to UDCA in primary biliary cirrhosis patients, few studies have attempted to identify factors that predict response to oral litholytic therapy in GB stone patients^14,15^.

The interaction between the gut microbiome and bile acids is closely coordinated by the bile acid-gut microbiome axis, suggesting that changes in bile acid composition directly influence the gut microbial environment. Recently, much research has been conducted on the gut microbiome, especially its role as a prognostic/predictive biomarker^16,17^. Grigor’eva et al. concluded the presence of *Helicobacter pylori* (*H. pylori*) infection and other gut microbiome components, such as *Clostridium*, *Bifidobacterium*, *Peptostreptococcus*, *Bacteroides*, *Eubacterium*, and *Escherichia coli*, contribute to GB stone formation and affect the development of gallstone disease complications. Georgescu et al. reported that patients with GB stone disease exhibited alterations in the abundances of several functional bacterial species, that is, reductions in abundances of butyrate, lactate, acetate/propionate, and methane producers, and mucin degrading bacteria, and reductions in microbiome biodiversity indices^17^. Furthermore, studies suggest that UDCA or CDCA treatments may impact gut microbiome composition and diversity. However, no research study has examined the effects of UDCA/CDCA on the gut microbiome and how these effects might be utilized to treat GB stones. Moreover, no prognostic or predictive biomarker has been identified for UDCA/CDCA treatment in gallstone patients.

This preliminary prospective study was undertaken to document and compare microbiome changes in patients that respond or do not to UCDA/CDCA treatment and to explore the potential use of the gut microbiome as a prognostic/predictive biomarker of response to UDCA/CDCA treatment.

## Methods

### Participants and study drug

This single-center, prospective, preliminary study was conducted from May 2021 to April 2022 with participants enrolled from Inha University Hospital. Eligibility criteria included: (1) Age above 18 years, (2) Detection of GB stones via abdominal ultrasonography, (3) No GB stones evident in standard abdominal X-ray imaging, (4) Being asymptomatic, and (5) No prior use of UDCA/CDCA. Exclusion criteria were: (1) Diagnosis with complicated GB stones, (2) Ongoing chronic kidney diseases, (3) Abnormal liver function test results, (4) A history of any cancer, (5) Use of antibiotics or probiotics within the preceding 2 months, (6) Symptoms indicative of gastrointestinal obstruction or inflammatory bowel disease, and (7) Bacterial diarrhea within the last 6 months. The control group comprised individuals who showed (1) Normal kidney and liver function test results, (2) Absence of hepatitis B/C virus antigens, (3) No cancer history, and (4) No use of antibiotics or probiotics in the 2 months prior to the study. The study was an open-label trial employing UDCA/CDCA capsules (CNU®; Myungmoon Pharmaceutical Company, Seoul, South Korea), each containing 114 mg of UDCA and CDCA. Participants were instructed to take two capsules each day: one with their breakfast and another in the evening with dinner, for a duration of 6 months.

### Patient evaluations

Participants were instructed to schedule follow-up appointments at the outpatient department 3 and 6 months following the initiation of treatment. At the beginning and during each subsequent visit, we conducted physical assessments and monitored for symptoms such as nausea, digestive discomfort, diarrhea, and abdominal pain. Collection of stool samples was done initially and then at the 3-month follow-up. We evaluated the levels of C-reactive protein (CRP), white blood cells (WBCs), aspartate aminotransferase (AST), alanine aminotransferase (ALT), alkaline phosphatase (ALP), total bilirubin (T.Bil) and γ-glutamyl transferase (r-GTP) at the start of the study and again at the 6-month mark. To assess the changes in the volume of GB stones, abdominal ultrasonography was performed during these visits. Based on the results of the ultrasonography, participants were categorized into either the responder or non-responder groups, as defined in the “The outcome definitions” section below.

### The outcome definitions

The volume of GB stones was determined by measuring the radius (r) of the largest stone identified through abdominal ultrasonography, with each stone assumed to be a sphere, and calculated using the formula 4/3π × r^3^. The dissolution rate was quantified as the percentage decrease in the volume of the GB stones. Complete dissolution is characterized by the total disappearance of the GB stone in the follow-up abdominal ultrasonography conducted six weeks after the initiation of treatment, while partial dissolution is identified by a reduction in stone size exceeding 50%. The response to therapy was assessed based on either complete or partial dissolution of the stones. A reduction in stone volume ranging between 0 and 50% was categorized as no meaningful response.

### DNA extraction from fecal samples

Stool samples were gathered prior to the start of UDCA/CDCA therapy and again three months into the treatment. These samples were initially kept at a temperature of − 80 °C before their dispatch to Bioeleven Co., Ltd. in Seoul, Korea, for comprehensive DNA sequencing and analysis. The process began with centrifuging the samples at 5000×*g* for 5 min at ambient temperature, followed by their suspension in 500 µL of cetyltrimethylammonium bromide solution as per the guidelines provided by the manufacturer. The extraction of DNA was conducted using the Maxwell® RSC PureFood GMO and Authentication Kit by Promega (Madison, WI, USA). To measure the bacterial DNA concentrations, we utilized a UV–Vis spectrophotometer (NanoDrop 2000c; Thermo Fisher Scientific, Waltham, MA, USA) along with the QuantiFluor® ONE dsDNA System from Promega. Until their use, all samples were preserved at a temperature of − 20 °C.

### PCR amplification of the V3–V4 region of the bacterial 16S ribosomal RNA (rRNA) gene

Amplification of the bacterial 16S rRNA gene’s V3–V4 variable region was achieved through a biphasic PCR method for 25 cycles at 55 °C. In summary, the PCR utilized a set of two primers: the forward primer was 5ʹ-TCGTCGGCAGCGTCAGATGTGTATAAGAGACAGCCTACG-GGNGGCWGCAG and the reverse primer was 5′-GTCTCGTGGGCTCGGAGATGTGTATAAGAGACA-GGACTACHVGGGTATC-TAATCC-3′. The PCR products underwent evaluation using 2% agarose gel electrophoresis. The 16S rRNA libraries were then purified with magnetic beads (AMPure XP), in line with the specifications given by Beckman Coulter, Wycombe, UK. To verify the purity of the samples, a Bioanalyzer 2100 (Agilent, Santa Clara, CA, USA) was employed. The second phase of the PCR involved attaching Illumina Nextera barcodes to the products of the initial PCR using i5 forward and i7 reverse primers. These amplified products were then purified following the same procedure as the initial round. DNA quantification was carried out using the QuantiFluor® ONE dsDNA System (Promega), and the Bioanalyzer 2100 was again used for assessing the quality of the samples. The amplified 16S rRNA gene and the prepared library, developed using this two-step PCR method, were sequenced using the MiSeq v3 Reagent Kit from Illumina, Inc.

### Data analysis

The sequencing data of 16S rRNA were analyzed utilizing the QIIME2 pipeline (version 2022.11)^18^. Initially, raw sequencing reads were subjected to quality control and trimming of bases with low quality scores (below 30) using Trimmomatic v0.39^19^. Deblur was used for denoising. The DNA sequences were subsequently organized into amplicon sequence variants (ASVs) through reference-based clustering, employing the Greengenes rRNA database (release 138) for reference. To discern the species richness and variations in microbial communities, both alpha and beta diversity metrics were computed using QIIME2. The diversity within the microbial population, indicated by the abundance of ASVs, was utilized to assess alpha diversity. This diversity was evaluated using three different indices: observed ASVs, Dominance D, and Shannon indices. In contrast, beta diversity was used to measure the compositional differences among ASVs across phylogenetic trees. For this, weighted Bray–Curtis distance matrices were generated from the anticipated metagenomes using QIIME and were examined through ANOSIM (analysis of similarities) with 9999 permutations. The data underwent statistical analysis employing Student’s-t-test or Repeated Measures Analysis of Variance (ANOVA) within R software version 4.2.1. The threshold for statistical significance was established at a *p*-value of less than 0.05.

### Metabolic pathway analysis

For the prediction of metagenomic functionalities, we employed PICRUSt2, a tool for Phylogenetic Investigation of Communities by Reconstruction of Unobserved States, integrated as a plugin in QIIME2. This tool facilitated the determination of the relative contributions of different KEGG (Kyoto Encyclopedia of Genes and Genomes) categories. Furthermore, within QIIME2, the MetaCyc pathways were standardized and subsequently subjected to in-depth analysis using the STAMP^20^. Additionally, the Longitudinal plugin was utilized to perform differential abundance analysis, focusing on various bacteria and metabolic pathways that were of particular interest in the study.

### Ethics statement

The research protocol was granted ethical approval by the Institutional Review Board at Inha University Hospital (Approval Reference: INHAUH 2021-03-051). Prior to the initiation of the study, all participants provided their written informed consent. The study was conducted in accordance with Good Clinical Practice guideline and the Declaration of Helsinki.

## Results

### Study subjects and GB dissolution rates after UDCA/CDCA treatment

Fifteen subjects with GB stones were initially included in this study, but two patients withdrew before completing 6 months of treatment. The demographic and clinical characteristics of the participants and controls are shown in Table 1 and Table S1. Of the 13 remaining patients, 8 responded to oral UDCA/CDCA treatment; 4 achieved complete stone dissolution and 4 partial dissolution. The other 5 patients had no meaningful response (n = 3) or increased GB stone volume (n = 2). 62% of patients showed a response to UDCA/CDCA treatment and the complete dissolution response rate was 31%. Based on treatment responses, participants were allocated to two groups, a responder group (the R group) and a non-responder group (the NR group). The 8 patients who responded to the treatment constituted the R group and the other five the NR group (Table 2).

**Table 1:** Demographic characteristics of study subjects.

Variables	Before UDCA/CDCA (n = 13)	After UDCA/CDCA (n = 13)	p*
Age (years)^§^	61 (40–72)	61 (40–72)	
Sex, female, n (%)^§^	10 (76.9%)	10 (76.9%)	
BMI, kg/m^2§^	23.5 (21.4–30.8)	23.5 (21.4–30.8)	
WBC (/uL)^§^	7410 (3560–12,980)	7120 (3750–9090)	0.42
AST (IU/L)^§^	23 (16–153)	19 (15–42)	0.15
ALT (IU/L)^§^	23 (9–247)	18 (13–39)	0.84
T.bil (IU/L)^§^	0.5 (0.3–1.1)	0.4 (0.3–1.1)	0.21
ALP (IU/L)^§^	84 (5–224)	67 (1–119)	0.18
PT (s)^§^	12.0 (11.4–12.5)	12.3 (11.8–13.3)	0.16

*BMI* body mass index, *WBC* white blood cell count, *AST* alanine aspartatetransferase, *ALT* alanine aminontransferase, *T.bil* total bilirubin, *ALP* alkaline phosphatase, *PT* prothrombin time.

^§^Median (range).

*p values were calculated using the *t*-test.

**Table 2:** Response to dissolution treatment with UDCA/CDCA.

Response	Number	%
Complete dissolution	4	31
Partial dissolution	4	31
Responders	8	62
No significant change	3	23
Increased	2	15
Non-responders	5	38

### Analysis of gut microbiomes by 16S rDNA sequencing

#### Impacts of UDCA/CDCA treatment on gut microbiome compositions

**Figure 1:**
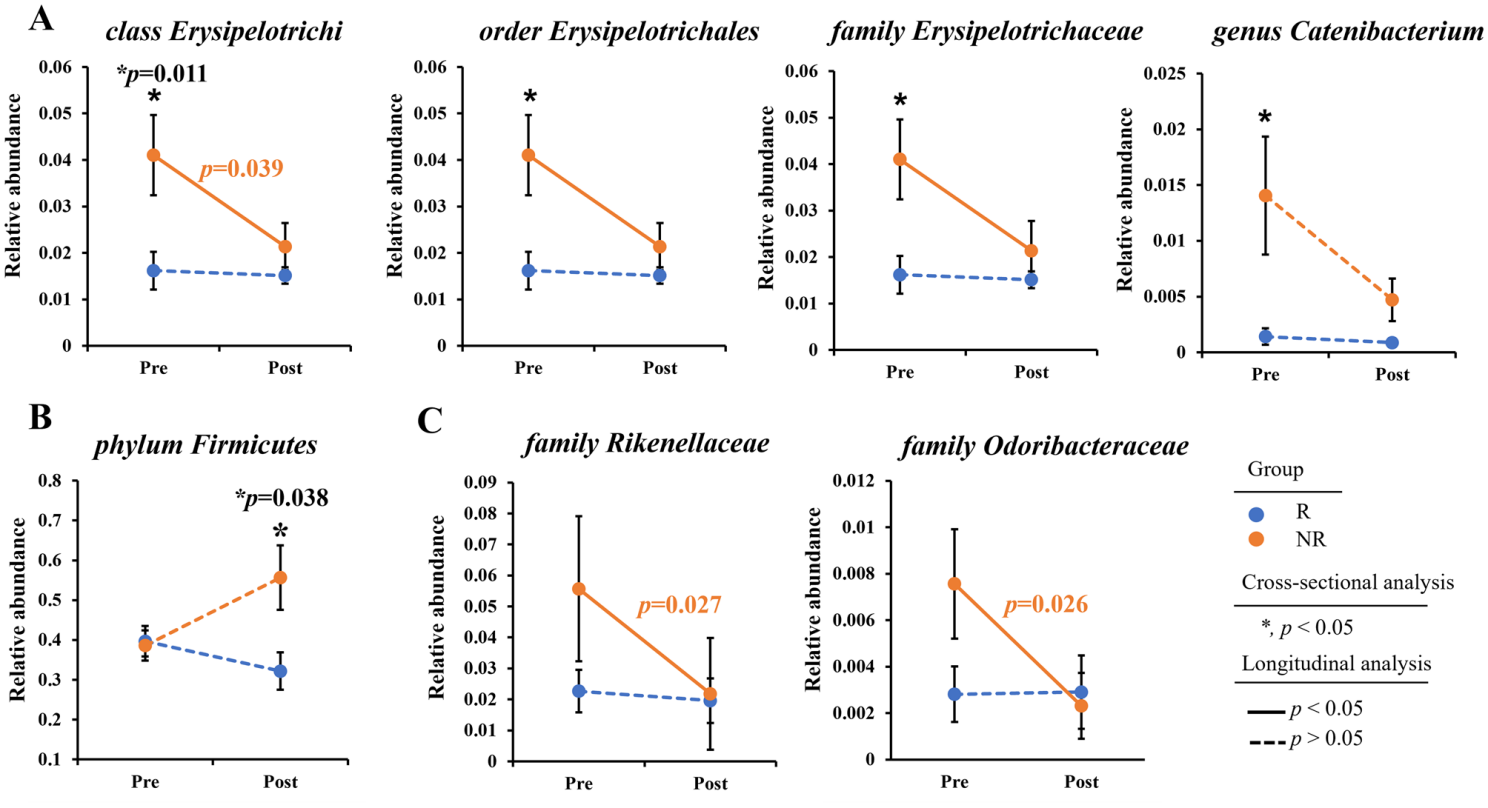
Bacterial abundance patterns of the R and NR groups before and after UDCA/CDCA treatment. A repeated measures analysis of variance (ANOVA) was performed to analyze the differences. (Blue dot: the responder group (R), orange dot: the non-responder group (NR), asterisk (*): significant in cross-sectional analysis (p < 0.05), solid line: significant in longitudinal analysis (*p* < 0.05), dash line: Not significant in longitudinal analysis (*p* > 0.05)). (**A**) Relative abundance of the *Erysipelotrichi* lineage. (**B**) Relative abundance of the *Firmicutes* phylum. (**C**) Relative abundance of the *Rikenellaceae* and *Odoribacteraceae* families.

**Figure 2:**
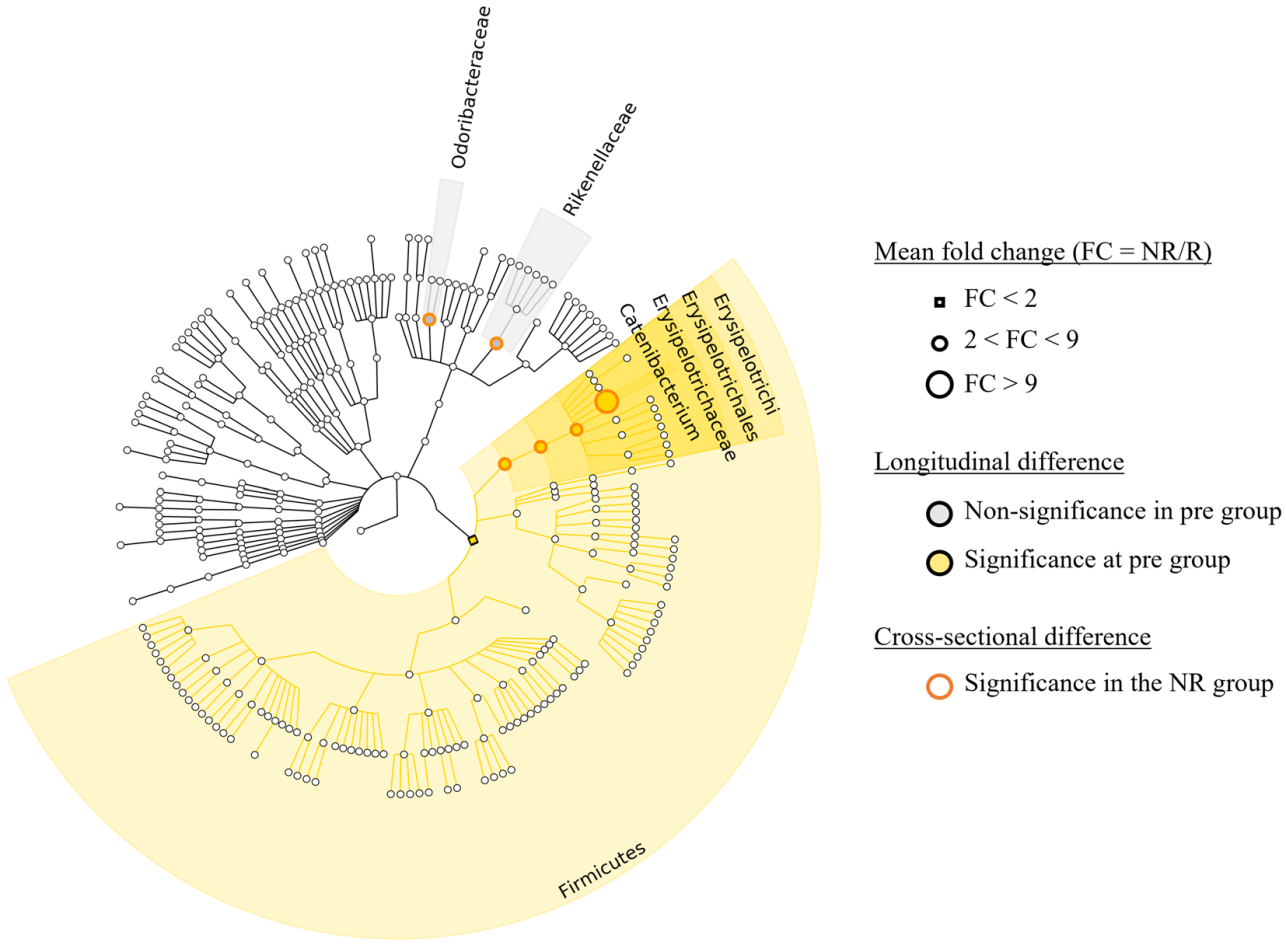
Cladogram. Cladogram showing significantly different abundances of bacterial taxa. (Small square: fold change (FC) < 2, medium circle: 2 < FC < 9, large circle: FC > 9, gray: not significant at pre group, yellow: significant at pre group, orange circle: significant at the non-responder (NR) group).

Bacterial abundance patterns were investigated before and after UDCA/CDCA treatment in the R and NR groups. Notably, the *Erysipelotrichi* lineage (class *Erysipelotrichi*, order *Erysipelotrichales*, family *Erysipelotrichaceae*, and genus *Catenibacterium*) was significantly more abundant in the NR group (*p* = 0.011). However, after 6 months of treatment, the abundances of these taxa decreased in the NR group (*p* = 0.039), resulting in no significant difference in abundance between the R and NR groups (Fig. 1A). At the phylum level, only *Firmicutes* showed significant differential abundance and was significantly more abundant in the NR group than in the R group after treatment (*p* = 0.038) (Fig. 1B). In addition, the abundances of the *Rikenellaceae* and *Odoribacteraceae* families were significantly reduced by treatment in the NR group to near those observed in the R group after treatment (*p* = 0.026 and 0.027, respectively) (Fig. 1C). The cladogram produced displays the bacteria with significant differences in abundances as identified by our analyses (Fig. 2).

#### Alpha and beta diversities

**Figure 3:**
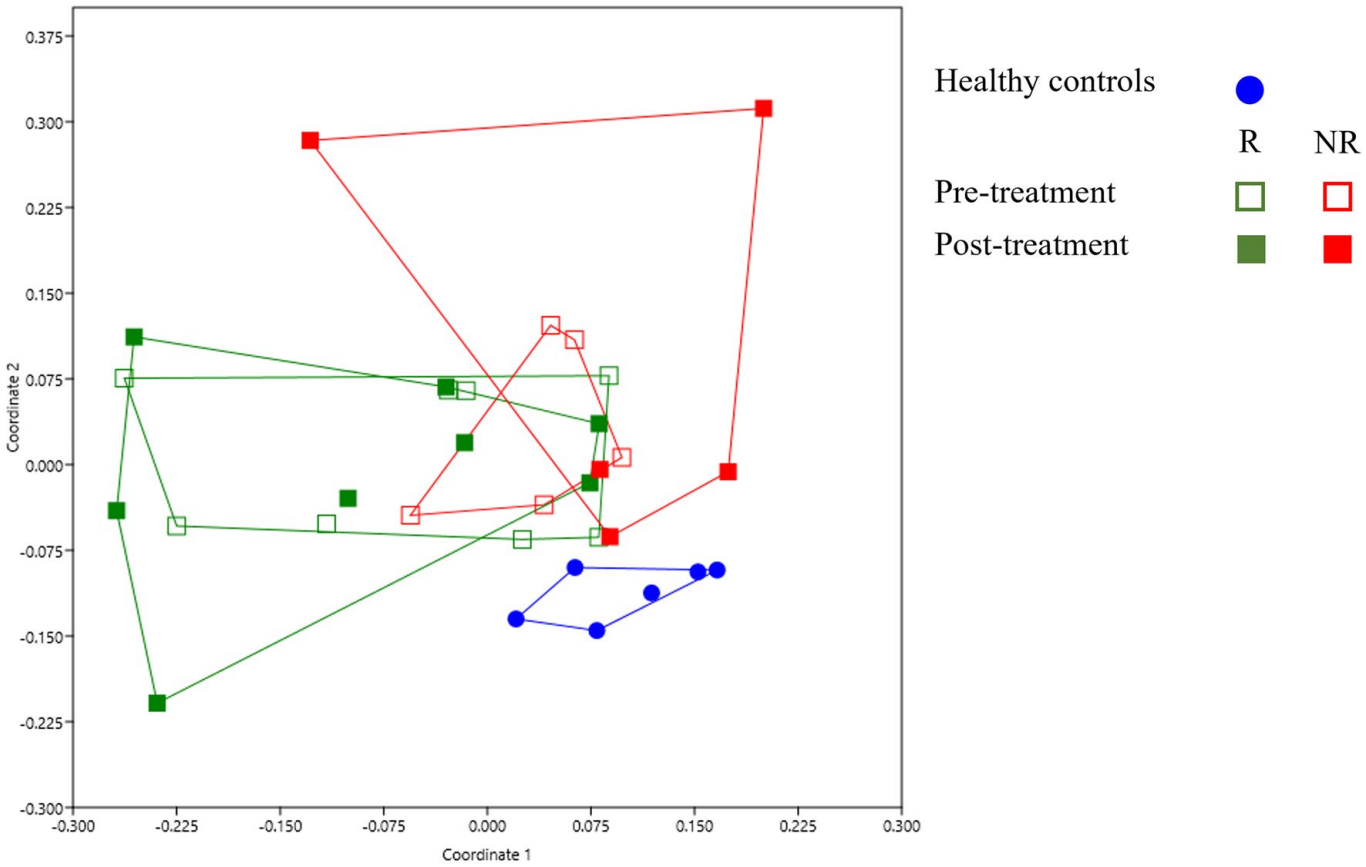
Beta diversity (non-metric multidimensional scaling (NMDS) plot). Group beta diversity analysis results determined using weighted Bray–Curtis distance matrices and the ANOSIM with 9999 permutations. (Blue dot: healthy controls, hollow green square: the pre-treatment responder group (R), solid green square: the post-treatment responder group (R), hollow red square: the pre-treatment non-responder group (NR), solid red square: the post-treatment non-responder group (NR)).

Beta diversity provides a valuable means of quantifying differences or similarities between microbial compositions across groups (Fig. 3). Beta diversity analyses revealed no notable differences in the gut microbiomes between R and NR groups before UDCA/CDCA treatment (pre-R vs. pre-NR, *p* = 0.425). In contrast, post-treatment groups (post-R and post-NR) exhibited significant differences in beta diversity (*p* = 0.029). Additionally, significant disparities were observed in the beta diversity among all groups and the six healthy controls (pre-R vs. controls, pre-NR vs. controls, post-R vs. controls and post-NR vs. controls, *p* = 0.019, 0.002, 0.005, and 0.043, respectively), clearly demonstrating distinct microbial compositions between GB stone patients and healthy individuals.

**Figure 4:**
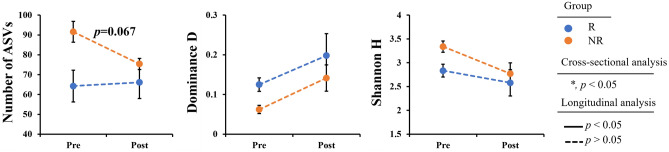
Alpha diversities (number of ASVs, Shannon and dominance D), a repeated measures analysis of variance (ANOVA) was performed to analyze the differences (Blue dot: the responder group (R), orange dot: the non-responder group (NR), asterisk (*): significant in cross-sectional analysis (p < 0.05), solid line: significant in longitudinal analysis (*p* < 0.05), dash line: Not significant in longitudinal analysis (*p* > 0.05)).

Alpha diversity analysis revealed no significant difference between the pre-R and pre-NR groups (Fig. 4), and longitudinal analysis failed to detect a significant difference between the R and NR groups before or after UDCA/CDCA treatment.

#### Predicted microbial pathway analysis using PICRUSt

**Figure 5:**
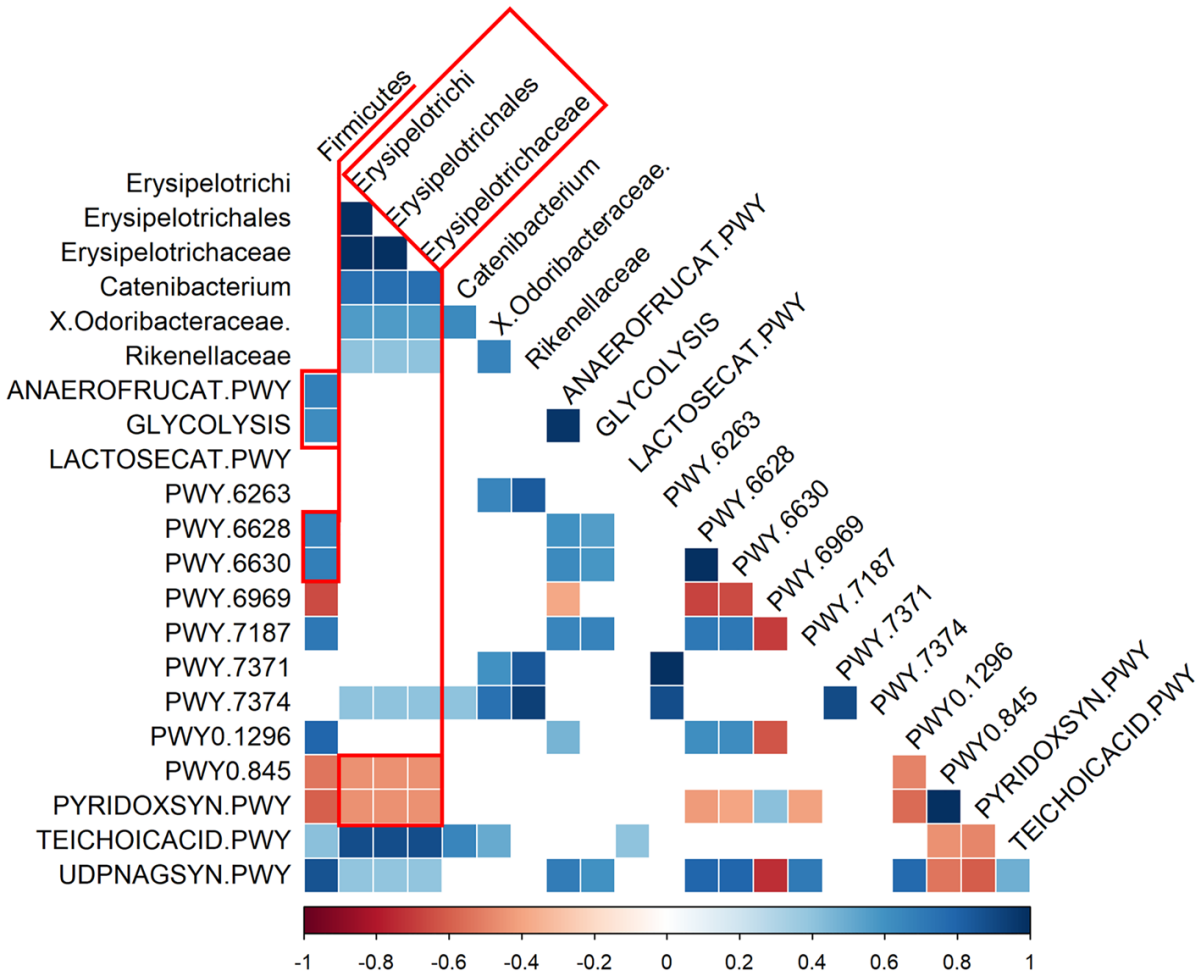
Integration of gut environmental factors. Pearson correlation heatmap of metabolites and gut microbiota. Red squares represent positive correlations, blue squares represent negative correlations, and white squares indicate no correlation.

The PICRUSt analysis results of predicted functional pathways in gut microbiome demonstrated that 15 predicted functional pathways are significantly associated with treatment and response states in GB stone patients, highlighting differences between groups based on these criteria (Fig. S1). Spearman correlation coefficients were calculated to explore the relationships between the relative abundances of bacterial families and their predicted functional pathways (Fig. 5).

Lactate (ANAEROFRUCAT-PWY and GLYCOLYSIS) and enterobactin biosynthesis pathways (PWY-6628 and PWY-6630) were upregulated by UDCA/CDCA in the NR group in parallel with increased *Firmicutes* abundance. Furthermore, vitamin B biosynthesis pathways (PWY0-845 and PYRIDOXSYN-PWY) seemed to be inhibited in patients with higher *Erysipelotrichi* lineage (class *Erysipelotrichi*, order *Erysipelotrichales*, family *Erysipelotrichaceae*) abundances. In addition, UDCA/CDCA treatment seemed to inhibit vitamin K biosynthesis pathways (PWY-6263, PWY-7371, and PWY-7374) in NR group, but there were no significant associations with specific microbiome.

## Discussion

This study shows that the gut microbiome has potential use for predicting the effectiveness of UDCA/CDCA treatment in patients with GB stones. Our results suggest that some bacterial lineages are susceptible to UDCA/CDCA treatment and that changes in gut microbiome composition may predict treatment response. Notably, a reduction in the abundance of *Erysipelotrichi* lineage was significantly associated with a favorable response to UDCA/CDCA, whereas an increase in *Firmicutes* phylum indicated no or poor response. These findings suggest that the abundances of these bacterial lineages might be useful positive or negative predictive biomarkers and that patients with high *Firmicutes* abundances may benefit from alternative treatments like cholecystectomy.

We found UDCA/CDCA treatment significantly decreased the abundance of *Erysipelotrichi* lineage in patients that achieved a favorable response. This lineage contains bacteria in the phylum *Firmicutes*, which is common in the gut microbiome. The role of the *Erysipelotrichi* lineage has not been extensively studied and therefore is largely unknown. However, some studies have suggested that *Erysipelotrichi* is linked to several diseases, particularly metabolic disorders and obesity. Kaakoush reported strong evidence linking *Erysipelotrichaceae* (a family of *Erysipelotrichi*) to metabolic disorders, including obesity and inflammatory bowel disease^21^, and it was suggested in another study that *Clostridium ramosum* (also a member of *Erysipelotrichi*), may promote obesity and play a role in the pathogenesis of obesity^22^. Interestingly, in our study, the relative abundance of *Erysipelotrichi* lineage before treatment was higher in the pre-NR group than in the pre-R group, and analysis showed a high *Erysipelotrichi* lineage abundance predicted lack of response to UDCA/CDCA treatment. Furthermore, we found that after 6 months of UDCA/CDCA treatment, the abundance of *Erysipelotrichi* lineages (class *Erysipelotrichi*, order *Erysipelotrichales*, family *Erysipelotrichaceae*, and genus *Catenibacterium*) in the NR group fell to the level in the R group, which suggests UDCA/CDCA ameliorated *Erysipelotrichi* lineage-related dysbiosis in the NR group (Fig. 1A). Moreover, a significant difference was observed between the beta diversities of the post-R and post-NR groups (*p* = 0.0289), indicating substantial variations in microbial compositions after the 6-month treatment period. These results show that, even after treatment, the R and NR groups possessed significantly different microbial profiles and suggest that higher doses of UDCA/CDCA or longer-term treatment might improve treatment response in these refractory patients. Therefore, we suggest that the effects of different UDCA/CDCA doses and treatment durations be subjected to further study.

The study also showed that increased levels of *Firmicutes* phylum might indicate UDCA/CDCA treatment failure in GB stone patients. The *Firmicutes* phylum is one of the major bacterial groups found in the human gut microbiome and has been the subject of much research in recent years^23^. The role of this phylum in human health is complex and not fully understood, but *Firmicutes* has been implicated in a variety of health outcomes, both positive and negative. On the positive side, some studies have suggested that *Firmicutes* may play a role in energy metabolism and weight regulation. For example, some have reported that obese individuals tend to have higher *Firmicutes* levels and suggested that reducing *Firmicutes* might help weight loss^24,25^. On the negative side, *Firmicutes* has also been associated with colorectal cancer, inflammatory bowel disease, and other digestive disorders^26^, and some studies indicate that gut microbiome imbalances, such as the overrepresentation of *Firmicutes*, might contribute to these conditions by disrupting the delicate balance of gut microbes. Further research is needed to understand the exact roles of *Firmicutes* and to determine the best approach for managing this phylum in the gut microbiome.

UDCA/CDCA treatment in our analysis was also predicted to impact the production of various metabolites in the gut microbiome, such as lactate and enterobactin, through several metabolic pathways, including ANAEROFRUCAT-PWY, GLYCOLYSIS, PWY-6628, and PWY-6630, and these metabolites might influence vitamin B and vitamin K biosynthesis and contribute to treatment failure. Specifically, patients who responded poorly to UDCA/CDCA treatment and an increased abundance of *Firmicutes* exhibited parallel increases in lactate and enterobactin, which implies a shift in the metabolic profile of the gut microbiome in NR patients. Lactate is a common metabolic end-product in many microorganisms, and high levels of lactate in the gut have been linked to several diseases, including inflammatory bowel disease. On the other hand, enterobactin is a siderophore produced by many *Enterobacteriaceae* species that is involved in iron acquisition. Furthermore, high levels of enterobactin have been linked to the increased virulence and antibiotic resistance of some bacteria. Our findings suggest that the observed increases in *Firmicutes* abundance and lactate and enterobactin in NR patients treated with UDCA/CDCA play roles in the mechanism of treatment failure. Further investigations are required to increase understanding of the relationship between the gut microbiome and oral litholysis treatment.

Our study has several limitations that should be addressed by future studies. First, the sample size of our study was small, which limits the generalizability of our findings. Second, although we investigated the relationship between treatment response and the gut microbiome and metabolic pathways in GB stone patients, we did not assess other metabolic aspects measurable by blood analysis. Therefore, additional analytical methods, such as blood or serum analysis, should be included in future studies to investigate associations between gut microbiome composition, bile acid metabolism, and other metabolic activities. Third, our use of PICRUSt2, which is based on analyzing short DNA sequences, limits our ability to accurately identify the specific functions of different bacterial strains, as it only provides limited genetic information. Overall, while the study provides valuable insights into the relationships between gut microbiome composition, bile acid metabolism, and treatment response in GB stone patients, further research is needed to confirm and build upon our findings.

In conclusion, this study shows that the UDCA/CDCA treatment results in gut microbiome and predicted bacteria pathway changes. Specifically, patients who responded poorly to treatment and exhibited a treatment-related increase in the abundance of the *Firmicutes* phylum exhibited a parallel increase in lactate and enterobactin related pathways, which suggests a shift in the metabolic profile of the gut microbiome in non-responsive patients.

### Supplementary Information

Supplementary Figure S1.

Supplementary Table S1.

## Data Availability

The datasets analyzed during the current study are available in the NCBI Sequence Read Archive repository under Accession Number SRP420350.

## Abbreviations

GB: Gallbladder
UDCA: Ursodeoxycholic acid
CDCA: Chenodeoxycholic acid
*H. pylori*: *Helicobacter pylori*
PICRUSt2: Phylogenetic Investigation of Communities by Reconstruction of Unobserved States
