# Supplementary figures and images for: The gut microbiome predicts response to UDCA/CDCA treatment in gallstone patients: comparison of responders and non-responders
Source: Sci Rep. 2024 Jan 30;14:2534. doi: 10.1038/s41598-024-53173-2 (PMC10828362; doi:10.1038/s41598-024-53173-2)

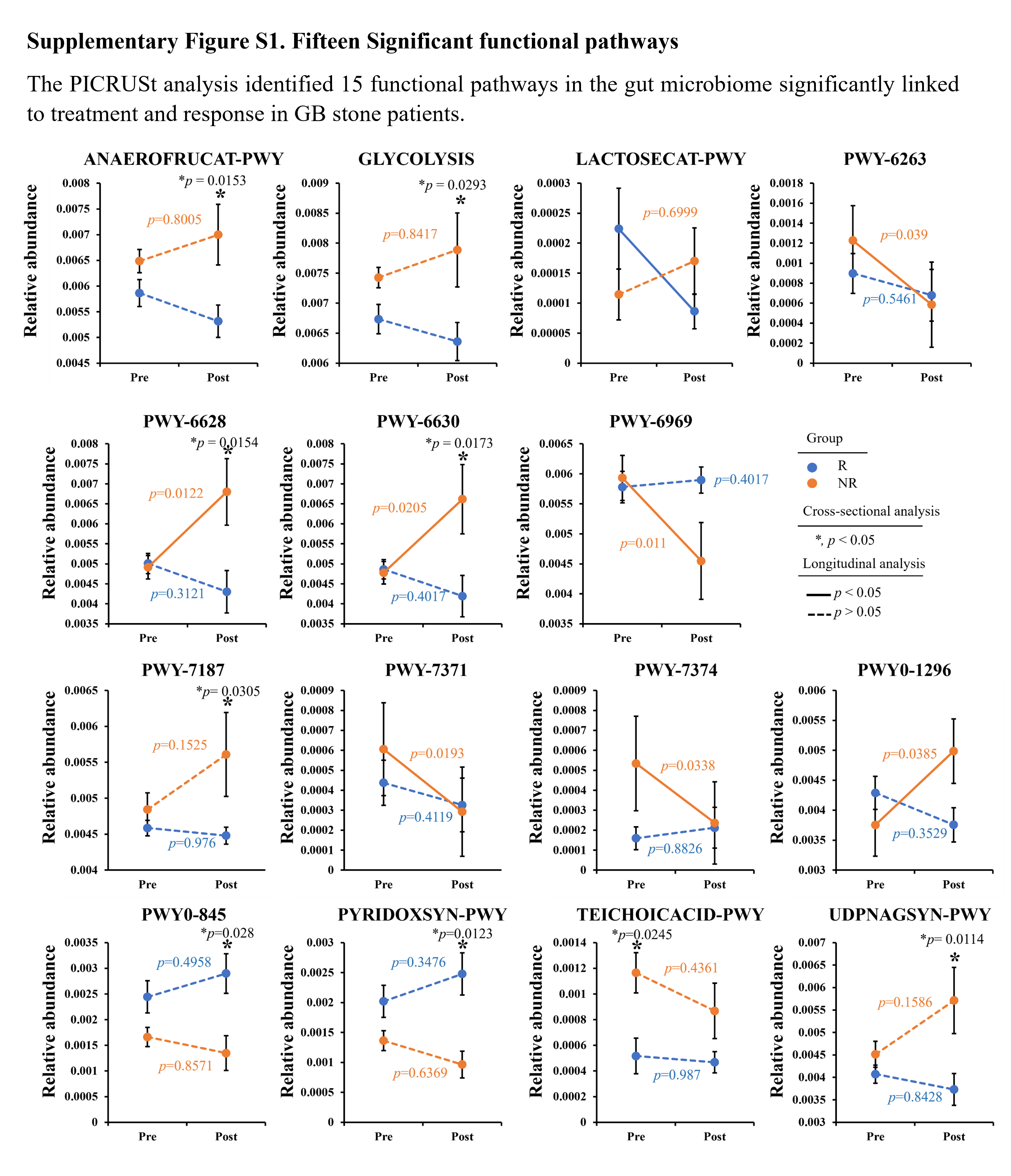

Supplement: Supplementary file 1 — Supplementary Figure S1. [file 41598_2024_53173_MOESM1_ESM.png]
