# Supplementary material for: The gut microbiome predicts response to UDCA/CDCA treatment in gallstone patients: comparison of responders and non-responders
Source: Sci Rep. 2024 Jan 30;14:2534. doi: 10.1038/s41598-024-53173-2 (PMC10828362; doi:10.1038/s41598-024-53173-2)
Supplement: Supplementary file 2 — Supplementary Table S1. [file 41598_2024_53173_MOESM2_ESM.docx]

**Supplementary Table S1. Demographic Characteristics of the Controls**

| **Sample number** | Sex | Age |
| --- | --- | --- |
| 1 | M | 45 |
| 2 | M | 43 |
| 3 | F | 53 |
| 4 | F | 60 |
| 5 | F | 64 |
| 6 | F | 77 |
